# An accidental household outbreak of paliperidone palmitate poisoning via pancake consumption in Lianyungang, China

**DOI:** 10.5365/wpsar.2019.10.1.005

**Published:** 2020-12-28

**Authors:** Tinglu Zhang, Zhentao Li, Peiliang Luo, Qingjun Sun

**Affiliations:** a Department of Emergency, Lianyungang Municipal Center for Disease Control and Prevention, Lianyungang, Jiangsu, China.; b Haizhou District Center for Disease Control and Prevention, Lianyungang, Jiangsu, China.; c Department of Paediatrics, Lianyungang No. 1 People’s Hospital, Lianyungang, Jiangsu, China.

## Abstract

**Introduction:**

At 11:20 on 26 May 2018, a physician from Lianyungang No. 1 People’s Hospital, China, reported that six family members were being treated in the hospital with symptoms from an unknown cause.

**Methods:**

A case series for a food poisoning investigation and an environmental survey were conducted. The patients and their relatives were interviewed in person with a questionnaire contained on a digital tablet, and an investigation of the patients’ home was conducted in the presence of police officers. Probable case and confirmed case were defined to serve as a basis for identifying additional cases. Confirmed cases were defined as those probable cases in which blood, stool or vomitus specimens tested positive for paliperidone palmitate and/or its metabolites. A descriptive analysis was performed. Follow-up by telephone was conducted four months later.

**Results:**

There were six probable cases. The median age was 35 years (range: 5–76 years). The attack rate was 100% (*n* = 6/6) of persons who consumed a family dinner, and the hospitalization rate was also 100% (*n* = 6/6). The median period between exposure and symptom onset was two hours. The main symptoms included vomiting, nausea, drowsiness, dizziness and severe abdominal pain for adults, and vomiting and severe lethargy for children. An 8-year-old girl further showed changes in the ST segment of her electrocardiogram, and a 5-year-old boy showed QT prolongation. The poisoning substance was suspected to be paliperidone palmitate based on the patients’ symptoms and epidemiological findings.

**Discussion:**

We investigated the household food poisoning outbreak through epidemiological analysis and an environmental investigation and determined that it was caused by paliperidone palmitate. The source of the paliperidone palmitate was found to be aluminium containers, taken home by the eldest son who worked at a pharmaceutical company. The containers were sent to a drug disposal centre, and the pharmaceutical company was required to enhance the regulation on the pharmaceutical waste materials to prevent drug poisoning events. By the end of September 2018, the six patients recovered and were released from the hospital, and they did not show any clinical sequelae in four follow-up visits.

At 11:20 on 26 May 2018, the staff at the Haizhou District Health Bureau received a call from a doctor in the Health Department informing them that six patients with symptoms from an unknown origin were being treated at the Liayungang No. 1 People’s Hospital, China. At that time, three epidemiologists and two laboratory personnel from the Lianyungang Municipal and Haizhou District Centers for Disease Control and Prevention were sent to that hospital to open an investigation.

## Methods

Epidemiological, laboratory and environmental investigations were conducted.

### Epidemiological investigation

A case series for a food poisoning investigation and an environmental survey were conducted in accordance with Chinese technical guidelines for epidemiological investigations of food safety incidents. (*1*)

We interviewed the patients and their relatives in person with a questionnaire contained on a digital tablet. The questionnaire included demographic information, clinical symptoms and treatments, and dietary exposure information over the previous 72 hours. Blood specimens and food samples were collected.

Probable cases were defined as members of the family who ate leftover fish, dry lettuce with sauce, scallion pancakes and rice porridge for dinner on 25 May 2018, and presented with acute gastroenteritis with at least one of the symptoms: vomiting, malaise and severe abdominal pain. Confirmed cases were defined as those who met the case definition for the probable cases, and whose blood, stool or vomitus specimens tested positive for paliperidone palmitate or its metabolites.

### Statistical analysis

We entered the data into a computerized database. The descriptive analysis included the distribution of onset dates and the process used to make the food. In addition, the attack rate (the number of cases divided by the number of family members who ate the scallion pancakes) and the hospitalized rate (the number of hospitalized cases divided by the number of cases) were calculated.

### Laboratory investigation

Blood specimens were collected from probable cases and sent to the municipal and provincial Center for Disease Control and Prevention laboratories and the pharmaceutical company laboratory. The dry lettuce (no other dishes from the meal were available) was collected (about 200 g) and sent to the Lianyungang Municipal Center for Disease Control and Prevention to test for pathogenic organisms.

### Ethical approval

This outbreak investigation was conducted by public health agencies as a part of their legally authorized mandate. It was, therefore, considered research with minimal risk and was exempted from ethical approval by institutional review boards.

## Results

### Case characteristics

The six patients being treated in hospital were members of a family that lived in Taiping Village, Haizhou District, Lianyungang, China. They included a 76-year-old man, a 63-year-old woman, a 35-year-old man, a 35-year-old woman, an 8-year-old girl and a 5-year-old boy.

The family’s dinner on 25 May 2018 included leftover fish from lunch, dry lettuce with commercially produced soybean sauce, homemade scallion pancakes and rice porridge (**Table 1**). The six patients ate dinner between 18:00 and 18:30. The eldest son went out for a haircut after dinner. Upon returning to the house, he found the five other members of his family in a lethargic state. He called emergency services, and ambulances soon arrived, taking the patients to the Emergency Department of Lianyungang No. 1 People’s Hospital for treatment.

**Table 1:** Types and approximate portions of food consumed by patients at dinner (18:00) on 25 May 2018

Sex	Age (years)	Dinner foods			
		Leftover fish from lunch (mouthfuls)	Dry lettuce with commercially produced soybean sauce (mouthfuls)	Pancake (g)	Rice porridge (bowls)
female	63	3	3	50	1
male	35	2	4	50	1
male	76	4	2	100	1
female	8	0	2	100	1
male	5	0	1	150	1
female	35	1	2	50	1

### Epidemiologic and clinical profiles

**Figure 1:**
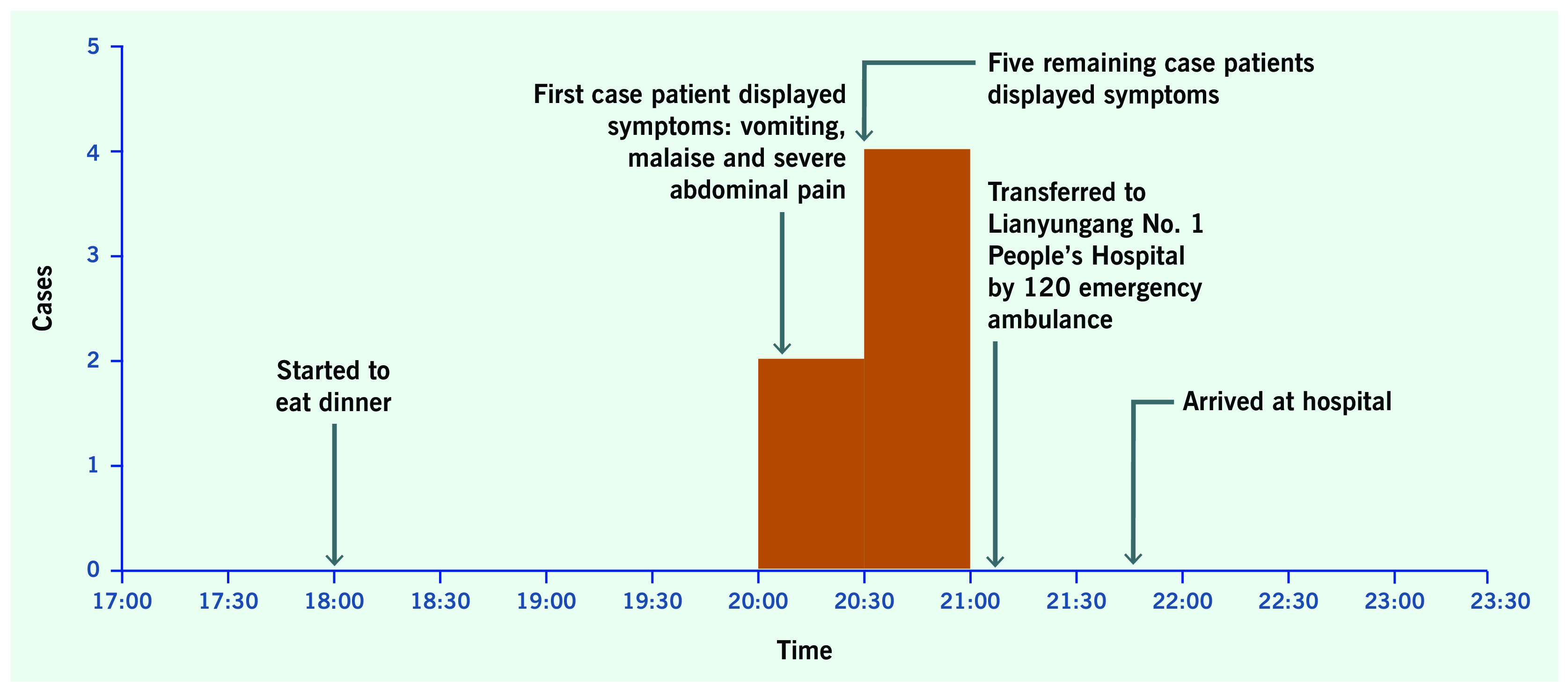
Distribution of six food poisoning cases in Taiping Village, Haizhou District, Lianyungang, China, on 25 May 2018

There were six probable cases. The first case displayed symptoms, at 20:10 on 25 May 2018; the five remaining cases displayed symptoms by 20:30. The median incubation period was two hours. The attack rate was 100% (*n* = 6/6) as shown in **Fig. 1**, and the hospitalized rate was 100% (*n* = 6/6).

[**insert** Figure 1]

All four adult case patients reported dizziness, drowsiness, malaise, nausea, severe abdominal pain and vomiting. Coffee-ground vomitus was not reported, indicating that gastric bleeding had not occurred. The main symptoms and signs exhibited by both children included contracted pupils, severe lethargy (one child was in a coma for two days, and the other for four days), tachycardia and vomiting, but they were otherwise haemodynamically stable. The girl showed ST segment changes in her electrocardiogram, and the boy displayed prolongation of the interval between the Q wave and the T wave (QTc prolongation).

### Laboratory

Blood specimens of all six case patients were collected and sent to the municipal and provincial Center for Disease Control and Prevention laboratories, but those laboratories did not have the capacity to test for paliperidone (as suspected from environmental investigation, see below). The dry lettuce tested negative for pathogenic organisms. Stool and vomitus specimens were not collected.

### Treatments

At 21:44 of 25 May 2018, the six patients arrived at Lianyungang No. 1 People’s Hospital by ambulance. The four adults were quickly transferred to the Emergency Department, and the two children were transferred to the Paediatrics Department. All six case patients were admitted to intensive care units and received supportive treatment with gastric lavage for presumed poisoning, followed by intravenous fluid infusion. They received diuretics to facilitate excretion of the paliperidone palmitate and its metabolites once they were in a stable condition, and after environmental investigation and according to the product prescription by Janssen Pharmaceutica N.V. The median length of hospital stay was 13 days (range: 12–15 days). Four follow-up visits were conducted by the end of September 2018; all six discharged patients reported no clinical sequelae.

### Food-making process investigation

Epidemiologists spoke with the 63-year-old’s niece, who reported that the grandmother had mixed a handful of white, tasteless “starch” from an aluminium container into the flour when making scallion pancakes on the afternoon of 25 May 2018. The epidemiologists visited the family home to investigate on the morning of 27 May 2018. Two aluminium containers with lids were found in the kitchen; there was a white substance at the bottom of each aluminium container. The containers were labelled “paliperidone palmitate.” These aluminium containers had been abandoned in a warehouse of a pharmaceutical company in Lianyungang, China, after most of the powder stored in the containers had been used. The grandmother’s eldest son worked for the company and had taken two aluminium containers from the warehouse, without permission, to use for storing items.

**Figure 2:**
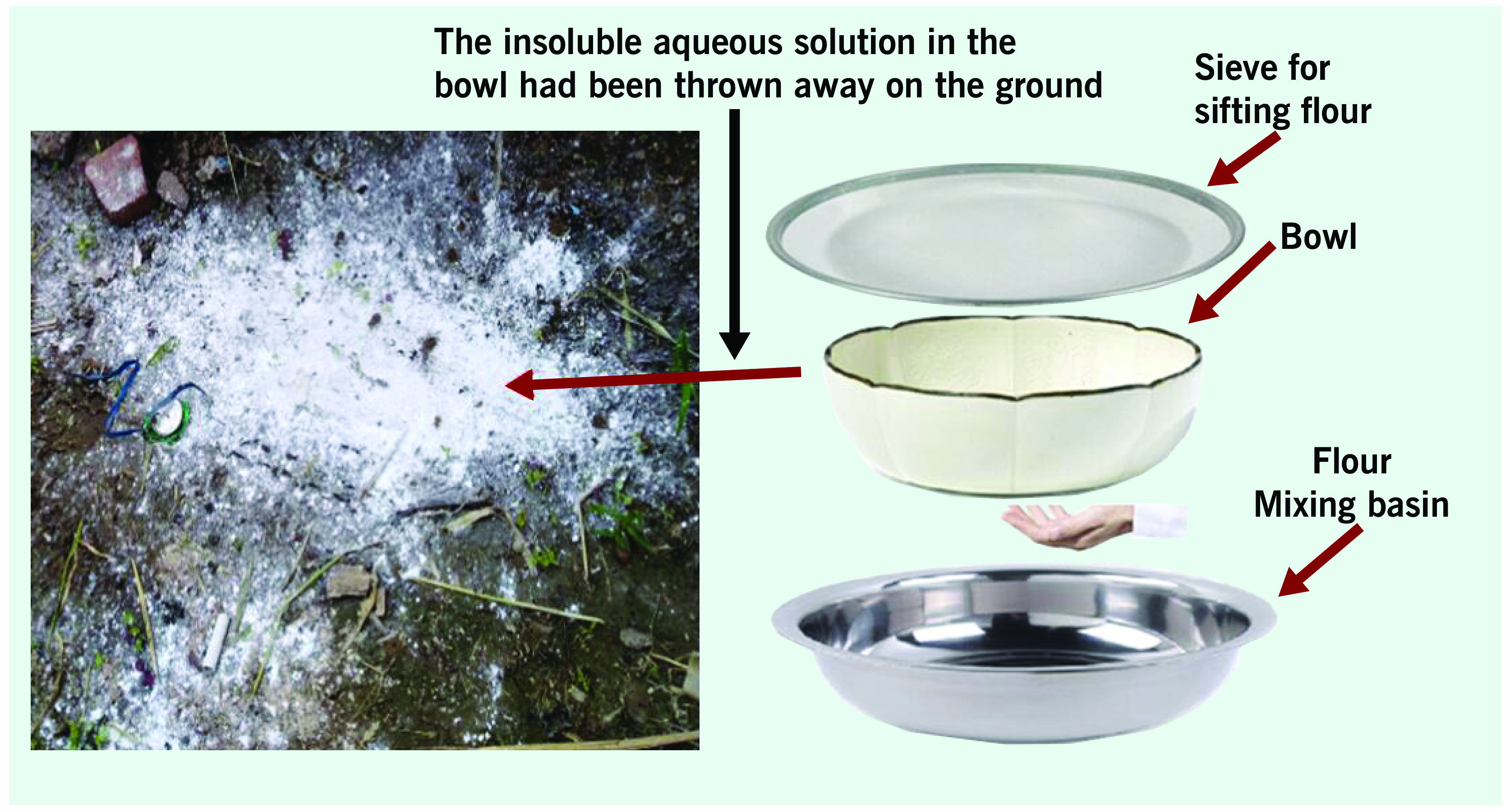
The insoluble “starch” solution was thrown away on the ground after sifting

On the morning of 27 May 2018, the grandmother described her food-making process to the epidemiologists. She told them that she took some white powder from an aluminium container that her son had brought home from the pharmaceutical company. She said he had told her that the white powder left in containers might be “starch.” She used a sieve over a flour-mixing basin to sift any impurities from what she thought was starch on the afternoon of 25 May 2018, then mixed the filtered white powder with water in a bowl. Because paliperidone palmitate is insoluble in water, the grandmother threw the contents of the bowl, including possibly 20 g of powder, on the ground. But there was still a little of what she believed to be starch (potentially 5 g) sprinkled into the flour-mixing basin during the sifting (**Fig. 2**). Finally, she used the flour-mixing basin to mix the wheat flour (about 500 g), and baked the scallion pancakes as a staple food for dinner.

[**insert** Figure 2]

### Control measures

The following public health control measures were implemented following the food poisoning outbreak and discovery:

Police officers sealed the aluminium containers and other related articles for safekeeping in the patients’ home, and then transferred them to the drug disposal centre for future elimination at the Lianyungang Public Security Bureau. None of the contaminated scallion pancakes remained.
Clinicians were informed of the discovery of paliperidone palmitate in the patients’ food, to inform treatment decisions”
The local Food and Drug Administration immediately launched an investigation into the food poisoning outbreak. It required the pharmaceutical company implicated to strictly enforce regulations on the destruction of expired materials or medicines in pharmaceutical production and storage and to standardize and update protocols for destroying those wastes to prevent drug poisoning events.

## Discussion

We describe a household food poisoning outbreak caused by paliperidone palmitate that accidentally contaminated flour that was incorporated into scallion pancakes consumed by a family in Taiping Village, Haizhou District, Lianyungang, China, on 25 May 2018.

Although we could not confirm it by laboratory testing, the epidemiologic and environmental investigation support the conclusion that the patients were poisoned with paliperidone palmitate, used in preparing the pancakes consumed by the family admitted to the Emergency Department.

Paliperidone palmitate is an antipsychotic medication used for the acute treatment and maintenance of schizophrenia cases. Previously reported adverse events include dizziness and vomiting, which were very similar to symptoms of the six patients, except for severe abdominal pain in this event. (*2*-*13*)

The half-life of paliperidone palmitate is 25–49 days after a single oral dose of 25–125 mg, according to the product prescription by Janssen Pharmaceutica N.V.

In this outbreak, the young girl showed ST segment changes in her electrocardiogram, and the young boy displayed prolongation of the interval between the Q wave and the T wave (QTc prolongation). Some studies have shown that prolongation of the QT intervals remains a concern with the use of antipsychotics. (*14*) While others have reported no evidence of clinically significant QTc prolongation with paliperidone palmitate at doses up to 100 mg equivalent.

When four follow-up visits for the discharged patients were conducted by the end of September 2018, all six discharged patients reported no clinical sequelae.

### Limitations

The laboratories did not have the capability to test for paliperidone palmitate.

## Conclusion

We reported a household food poisoning outbreak that is suspected to have been caused by paliperidone palmitate that was accidentally incorporated into scallion pancakes, according to the clinical symptoms of six patients and the epidemiological findings.

It is suggested that the pharmaceutical company strictly enforce regulations on the destruction and disposal of pharmaceutical waste and expired materials. In addition, it is suggested the people not take home unknown and/or abandoned commodities and not to eat unknown food to prevent the food-related poisoning events.
